# Compensation preferences of home-based disabled beneficiaries in the long-term care insurance system in Guangzhou, China

**DOI:** 10.1093/heapol/czaf015

**Published:** 2025-04-01

**Authors:** Yi Qian, Yusupujiang Tuersun, Duqiao Li, Lan Luo, Yingdan Kou, Wenhao Yuan, Lehuan Li, Liancheng Zheng, Mengping Wu, Wenyu Wang, Jiangyun Chen

**Affiliations:** School of Health Management, Southern Medical University, No.1023-1063 Shatai Road, Baiyun District, Guangzhou City, Guangdong Province 510515, China; School of Health Management, Southern Medical University, No.1023-1063 Shatai Road, Baiyun District, Guangzhou City, Guangdong Province 510515, China; School of Health Management, Southern Medical University, No.1023-1063 Shatai Road, Baiyun District, Guangzhou City, Guangdong Province 510515, China; Hongshan Street Community Health Service Center, Huangpu District, No. 601, Guangxin Road, Huangpu District, Guangzhou City, Guangdong Province 518055, China; School of Health Management, Southern Medical University, No.1023-1063 Shatai Road, Baiyun District, Guangzhou City, Guangdong Province 510515, China; School of Health Management, Southern Medical University, No.1023-1063 Shatai Road, Baiyun District, Guangzhou City, Guangdong Province 510515, China; School of Health Management, Southern Medical University, No.1023-1063 Shatai Road, Baiyun District, Guangzhou City, Guangdong Province 510515, China; School of Nursing, Southern Medical University, No.1023-1063 Shatai Road, Baiyun District, Guangzhou City, Guangdong Province 510515, China; School of Nursing, Southern Medical University, No.1023-1063 Shatai Road, Baiyun District, Guangzhou City, Guangdong Province 510515, China; Department of Academic Affairs, Nanfang Hospital, Southern Medical University, 1838 North Guangzhou Avenue, Guangzhou City, Guangdong Province 510515, China; School of Health Management, Southern Medical University, No.1023-1063 Shatai Road, Baiyun District, Guangzhou City, Guangdong Province 510515, China; Institute for Hospital Management of Henan Province, No. 1 Longhu Zhonghuan Road, Jinshui District, Zhengzhou City, Henan Province, China, Zhengzhou 450003, China

**Keywords:** China, health policy, elderly, long-term care insurance system, discrete choice experiment

## Abstract

This study explores the preferences and willingness-to-pay of home-based disabled elderly individuals for long-term care insurance (LTCI) compensation mechanisms in Guangzhou, China, using a discrete choice experiment. The research aims to identify preferred compensation strategies, analyze heterogeneity in preferences, and provide recommendations for policy optimization. Using purposive and cluster sampling, 156 eligible participants were identified, with 96 completing the survey (response rate: 61.5.%). Disabled elderly individuals were defined based on activities of daily living assessments. A conditional Logit model was applied to analyze preferences, and subgroup analyses examined differences by education, gender, activities of daily living status, and caregiving arrangements.

Key findings include preferences for medical care over life care, family caregivers over professional ones, and cash subsidies over mixed or proportional reimbursement. Respondents were willing to pay an additional $21.60 for medical care and $25.26 for cash subsidies (1 USD = 7.3 CNY). The average out-of-pocket cost for LTCI services was $27.39 per session, with a sub-average cost of $16.44 for basic care services. Subgroup analyses revealed higher-educated individuals favored medical care, while lower-educated groups prioritized affordability. Severely disabled individuals preferred professional caregivers, such as registered nurses. This study highlights the need to expand medical care services, integrate flexible compensation models, and tailor policies to demographic differences. The findings provide evidence for optimizing China’s LTCI system and offer insights for aging populations in low- and middle-income countries.

Key messages

- Preferences for long-term care insurance (LTCI) compensation strategies. This study reveals the preferences of home-based disabled elderly individuals for LTCI compensation mechanisms. The findings show that respondents prefer medical care over life care, family caregivers over professional caregivers, and cash subsidies over mixed or proportional reimbursement. Additionally, respondents were willing to pay an additional $21.60 for medical care and $25.26 for cash subsidies (1 USD = 7.3 CNY).
- Population heterogeneity. Subgroup analyses highlight that factors such as education level, gender, activities of daily living status, and caregiving arrangements significantly influence preferences for LTCI. Higher-educated individuals preferred medical care, while lower-educated groups prioritized affordability. Severely disabled individuals preferred professional caregivers, such as registered nurses.
- Policy optimization recommendations. The study suggests that the current LTCI system faces challenges, including insufficient medical care services and inflexible compensation models. It is recommended to expand the range of medical care services, integrate flexible compensation models, and tailor policies to meet the demographic differences of various groups, thereby improving coverage and satisfaction among beneficiaries.
- Implications for China and low- and middle-income countries. The findings offer valuable insights for optimizing China’s LTCI system and provide implications for aging populations in low- and middle-income countries, helping inform future policy developments in these regions.

## Introduction

A rapid growth in the aging polpulation has become a global challenge, creating significant demand for medical and care services while imposing substantial financial pressures, particularly in the post-COVID-19 era (Xu et al. 2019). According to data from China’s Seventh National Population Census, the population aged ≥60 years has reached 264 million, accounting for 18.70% of the total population, while those aged ≥65 years number 191 million, making up 13.50% of the total population (National Bureau of Statistics: China National Center for Aging Studies 2019). China now has the largest population of people aged ≥65 years in the world and faces severe challenges related to advanced aging (Chen and Zhao 2023). Compared to other countries, aging in China is characterized by a large population base, the prevalence of ‘empty-nest’ families, the pace of population aging is more rapid, higher disability rates, and widespread chronic diseases (Xiang and Wang, 2021). Due to the widespread prevalence of chronic illnesses among the elderly, a considerable proportion of China’s large aging population is in a state of disability and unable to perform daily self-care (Xu and Chen 2019). The Fourth Survey Report on the Living Conditions of Urban and Rural Elderly in China estimated that the disabled and semi-disabled elderly population in China is ∼40.63 million, accounting for 18.3% of the elderly population; this figure is projected to grow to 61.68 million by 2030 and 97.50 million by 2050 (National Bureau of Statistics 2021). Moreover, the typical ‘4-2-1’ family structure in China—where one couple supports four elderly parents and one child—has further intensified caregiving pressures on families, driving rapid growth in the demand for long-term care services and insurance (Zhong and Yan 2023). This trend not only exacerbates the supply–demand imbalance in medical and care services but also highlights the urgent need for innovative policy tools, such as long-term care insurance (LTCI), to meet the growing demand for care.

Since the 1960s, many countries have gradually established and developed long-term care service systems, providing critical policy support to address the challenges of aging populations. Research indicates that such systems not only significantly alleviate the economic burden on families of disabled elderly individuals but also play an essential role in improving their quality of life and well-being (Atal et al. 2023). To proactively address the challenges posed by population aging, China launched a pilot LTCI program in 2016. Starting with 15 cities, the program expanded to cover 49 pilot cities nationwide by 2021, with >140 million participants (Chen and Zhao 2023). The Chinese LTCI system offers three primary forms of care services: institutional care, community care, and home care. However, compared to institutional care, the home care model aligns more closely with China’s traditional cultural concept of ‘raising children for old age’ and is better suited to the socioeconomic conditions of most families, making it the dominant form of LTCI services (Xu 2023).

Despite the government’s vigorous promotion of family- and community-based LTCI schemes, home care services in China still face numerous challenges. Research highlights that due to shortages of resources and weak infrastructure, home care services in China predominantly rely on domestic service agencies, resulting in relatively high costs. Additionally, the shortage of professional caregivers has led to generally low service quality (Sawamura et al. 2015). Furthermore, the LTCI system in China exhibits significant ‘fragmentation’ in both coverage and service content (Dai and Yang 2021), leaving the care needs of home-based disabled elderly individuals inadequately addressed, particularly in terms of the provision of formal and professional home care services.

Research shows that home-based disabled elderly individuals are among the primary service recipients of LTCI and represent the group with the most urgent care needs. Their demands are significantly influenced by multiple factors, including gender, age, education level, economic status, family support, and health condition, exhibiting a high degree of individualization and diversity (Aizaki and Nishimura 2008, Lancsar and Louviere 2008, Nieboer et al. 2010). However, existing research and practices in LTCI have yet to develop comprehensive compensation mechanisms tailored to the specific needs of this group. There is a lack of detailed, elderly-centered care service inventories and a well-defined, refined combination of care plans to address their unique requirements.

To better meet the personalized care needs of home-based disabled elderly individuals, this study focuses on the compensation mechanisms of LTCI by employing discrete choice experiments (DCEs) to explore their preferences for relevant personalized attributes. As an important research tool in health economics, DCEs enable a comprehensive measurement of the target groups’ preferences and influencing factors by simulating choice scenarios (Araña et al. 2008). By designing attributes and levels based on policy considerations, this study systematically investigates the key factors affecting the care choices of home-based disabled elderly individuals and evaluates the willingness-to-pay for various factors and levels. The findings of this study provide critical evidence for optimizing differentiated and refined compensation strategies within the LTCI framework.

DCEs have been widely applied in the health and care fields in recent years to evaluate individual preferences and willingness-to-pay for various aspects of products or services (de Bekker-grob et al. 2012). In the field of long-term care, DCEs have been used in countries such as Australia, Japan (Sawamura et al. 2015), Italy (Brau and Lippi Bruni 2008), and The Netherlands (Nieboer et al. 2010, Wang et al. 2022a) to study preferences for high-risk care services, long-term care facilities, and LTCI policies. These studies have provided valuable references for policy-making.

In China, DCE-based research on LTCI has also made significant progress in recent years. Representative studies include Wang’s analysis of the technical details of attribute design, offering methodological guidance for future research (Wang et al. 2021b). Other studies have focused on the preferences and willingness-to-pay of elderly residents for different LTCI attributes, revealing heterogeneity in demand and payment capacity, thus providing supportive data for policy-making (Ma et al. 2023a). Research from a demographic perspective has analyzed the influence of age, gender, and economic status on LTCI policy needs, highlighting the importance of population characteristics in policy design (Ma et al. 2023b). Additionally, preferences and willingness-to-pay for LTCI service attributes among community residents have been explored, offering empirical support for policy optimization (Wang et al. 2021a). Further studies have examined the feasibility of integrating DCE methods into LTCI research, evaluating the impact of key attributes such as waiting periods, maximum monthly benefits, and individual payment ratios on decision-making (Chandoevwit and Wasi 2020). Collectively, these studies provide crucial theoretical and practical insights for optimizing LTCI policy design.

These studies provide fundamental data for understanding the demand characteristics and beneficiary preferences within China’s LTCI system. However, most of the existing literature focuses on the general population, with limited research specifically targeting disabled elderly individuals, particularly home-based disabled beneficiaries. Furthermore, current studies often overlook home care, the dominant mode of long-term care in China, and lack alignment with practical policy implementation. There is also an absence of detailed investigations into specific compensation strategies. For instance, the coordination between family members as primary caregivers and professional care services has not been thoroughly examined, and the heterogeneity in willingness-to-pay as well as the adaptation of policies to stratified needs remain underexplored.

Against this backdrop, this study focuses on home-based disabled elderly individuals and employs DCE methods to analyze their preferences for LTCI compensation mechanisms. The research not only addresses their personalized care needs but also examines preference heterogeneity across dimensions such as education-level, gender, and activities of daily living (ADL) status. The objective is to provide empirical support for optimizing policy design, particularly by proposing targeted strategies for compensation under the home care model, thereby addressing gaps in the existing literature.

## Methods

### Definitions

#### Disabled elderly

In China, disabled elderly individuals are defined as those who, based on an ADL assessment, are unable to independently perform essential daily activities such as eating, dressing, and toileting. In this study, the term ‘disabled elderly’ specifically refers to older adults who meet the eligibility criteria for LTCI under the assessment procedures outlined in the Guangzhou Pilot Plan for LTCI. Specifically, after receiving an application, the local government assigns professional care personnel to evaluate the applicant’s level of disability. Subsequently, the assessment results are verified by relevant supervisory departments to confirm eligibility. Elderly individuals who pass this evaluation process are classified as ‘disabled elderly’ as defined in this study and commence receiving LTCI services (Guangzhou Municipal People’s Government 2019).

#### Home-based care

Home-based care refers to long-term care services provided primarily in the home setting, either by family members or professional caregivers, for disabled elderly individuals. These services encompass daily living support, such as bathing and feeding, as well as basic medical care. In China, due to the significant shortage of eldercare institutions and the influence of traditional values such as ‘raising children for old-age support’ and ‘family-based eldercare’, the majority of disabled elderly individuals opt for care within the family environment. This approach not only aligns with cultural norms but also helps reduce economic burdens on both society and families to some extent.

#### Local residents

Local residents are defined as individuals who have resided within the study area for at least 6 months and possess either local household registration (hukou) or a valid long-term residence permit. In China, LTCI is still in its pilot phase and has not yet been implemented nationwide. According to the pilot policy, only elderly individuals who meet the criteria for ‘local residents’ are eligible to undergo LTCI assessment and, upon passing, receive the associated benefits. This policy is designed to ensure that the resources of pilot areas are prioritized for elderly individuals who reside locally on a long-term basis, reflecting the localized nature of LTCI services.

### Sample and data collection

This study employed purposive sampling and cluster sampling methods to select the study site and participants. Specifically, in the Huangpu District of Guangzhou, communities with a higher concentration of disabled elderly individuals were identified through purposive sampling. Subsequently, cluster sampling was used to include eligible disabled elderly individuals from these selected communities as study participants. Guangzhou, as the capital city of Guangdong Province, is one of the first 15 pilot cities for LTCI in China, making it highly representative. As of the time of this study, 49 cities across the country had participated in the LTCI pilot program, covering >140 million people. Among these cities, Guangzhou has developed a flexible and decentralized LTCI implementation model, balancing social benefits with practical feasibility. Since the pilot program’s launch on 1 August 2017, Guangzhou has continuously adjusted and optimized its policy coverage and service content, accumulating extensive practical experience that serves as a reference for pilot programs in other regions.

The study site, the Hongshan Street Community Health Service Center in Huangpu District, is one of the key designated institutions in Guangzhou’s LTCI pilot program. The center is not only a demonstration facility for integrated medical and eldercare services in Guangdong Province but also a pilot unit for palliative care in Guangzhou. Its extensive service network includes a main center, four satellite sites, one nursing station, one eldercare center, and an embedded nursing home. With 320 staff members and 220 open beds, the center offers diverse services, including geriatric integrated medical care, rehabilitation therapy, and community-based home care for the elderly. As of the time of this study, the center had provided services to 156 LTCI beneficiaries, with >8000 service instances recorded.

According to health statistics from Huangpu District, ∼10% of the local elderly population met the eligibility criteria for LTCI, and the study participants represented 78% of this eligible group, ensuring high representativeness and coverage. In summary, the Hongshan Street Community Health Service Center, with its comprehensive infrastructure, extensive experience in the LTCI pilot program, and diverse service offerings, was an ideal site for this study. Its selection not only ensured the reliability and relevance of the study findings but also reflected broader trends in the implementation of LTCI in Guangzhou.

The inclusion criteria for the sample were: (i) aged ≥60 years; (ii) permanent resident of Guangzhou City (residing for ≥6 months); (iii) in a state of disability or recovery; (iv) living at home; and (v) currently receiving home-based long-term care insurance benefits during the study period. The exclusion criteria were: (i) non-local elderly; (ii) those with terminal illnesses and an expected lifespan of <12 months (the care needs of patients with terminal illnesses are often more focused on end-of-life care and palliative care); (iii) individuals with mental disorders; and (iv) elderly living alone and supported by government welfare known as ‘Five Guarantees’ households. (In China, individuals under the Five Guarantees Program are a special group receiving comprehensive social security directly provided by the government. Their care services and financial support are typically funded through government welfare programs rather than the LTCI system.)

Due to the complexity of the discrete choice questionnaire, our household survey began with scheduling visits to homes of disabled elderly individuals. During the in-home research, our investigators were paired one-on-one with nurses from institutions providing LTCI services to collaboratively conduct the survey. After completing the questionnaire with an investigator’s assistance, the investigator reviewed, collected, and retained contact information of each respondent for future reference, allowing researchers to correct survey data in a convenient and timely manner during data verification. For respondents with difficulty expressing themselves in Mandarin, or with other language impairments that hindered clear expression of their wishes, nurses from the LTCI who conducted home visits assisted in one-on-one questioning. With permission, the investigators recorded the conversations and noted down responses based on the subjects’ answers.

### DCE

The DCE is one of the classic methods for measuring stated preferences. The DCE assumes that a product or service can be described by multiple attributes, each of which can be defined at different levels. These attributes and levels are combined to form a questionnaire consisting of a series of choice sets. Respondents make selections within these choice sets, and statistical analysis of these selections can reveal the impact of attribute factors on the respondents’ willingness to choose and their preference degree for these attributes (Lancsar and Louviere 2008). The DCE enables respondents to consider multiple factors simultaneously, reflecting their own weighing process for each attribute.

### Determining attributes and levels

In this study, through a systematic literature review, we initially established ten potential attributes across three dimensions for inclusion in the experiment (see supplementary Table S1 in the online supplementary material). These attributes were: care content, per service cost, care personnel, frequency of care, compensation method, service period, service location, service institution, reservation method, and service time. The literature review included 104 studies on LTCI policies across 49 pilot cities in China and international studies on policy preferences, providing comprehensive insights into the key factors influencing respondents’ preferences for LTCI.

To further refine the attribute selection, we followed the World Health Organization DCE guidelines and previous research practices. Using qualitative research methods, we conducted interviews and surveys with experts in the public policy and LTCI fields. A purposive sampling approach was adopted to select experts, and they were invited to evaluate the importance of the ten attributes, propose supplementary factors, and define the levels for certain attributes. The survey results are detailed in supplementary Table S2, see online supplementary material.

Based on the frequency and importance ratings from expert feedback, we identified the five most critical attributes for inclusion in the experiment: care content, per service cost, care personnel, frequency of care, and compensation method. The final set of attributes and their corresponding levels are presented in Table 1.

**Table 1.** Characteristics and levels of inclusion in DCEs

Characteristic factor	Definition	Level
Content of care	Specialized orientation and main content of services provided by LTCI	(i) Life care is the mainstay (ii) Medical care
Average per capita cost	Payment acceptable to the insurance provider for each hour of service received	(i) Out-of-pocket < $100 (ii) Out-of-pocket 100–300 yen (iii)Out-of-pocket> 300 yen
Nursing staff	Personnel providing services to the demand side of long-term care insurance	(i) Family members (ii) Care worker (iii) Registered nurse
Frequency of care	Number of times the supplier provided long-term care insurance services to the demand side on a 1-week cycle	(i) 4 h, 7 times/week (ii) 1 h, 7 times/week (iii) 2 h, 1 time/week
Mode of compensation	Government-to-demand payments	(i) Mixed benefits (ii)Proportional reimbursement (iii) Cash subsidy

### Experimental design and questionnaire development

This study required participants to make choices among different levels of attributes and select their preferred compensation mode for LCTI. We identified 1 two-level factor and 4 three-level factors, resulting in a total of 3^4^ × 2^1^ = 162 different options for LTCI compensation modes. If these options were combined into choice sets in pairs, it would form 162 × 161/2 = 13 041 choice sets. Obviously, including all these choice sets in a choice test questionnaire is unfeasible. Therefore, this study employed an orthogonal design using SPSS. In the orthogonal design, the number of factors (attributes) and their level counts (levels corresponding to attributes) for inclusion in the experimental study were input into SPSS, which generated 16 options. The option with the most balanced level for one of the attributes was selected as the reference group, and the remaining 15 options were compared with it to form a choice set (World Bank 2013). Combinations with obvious advantages or disadvantages in the reference and comparison groups were excluded, resulting in the final 15 choice sets (Aizaki and Nishimura 2008). Considering the level of understanding of the choice tests, these 15 choice sets were divided into two parts, thus forming two questionnaires. The other parts of the two questionnaires were the same, only differing in the choice sets of the choice tests.

### Data analysis

The study data was organized in Excel according to the standards of DCE analysis. The discrete choice conditional Logit model and willingness-to-pay analysis were performed using Stata 17.0 while other analyses were carried out using SPSS21.0.

Descriptive analysis

General demographic characteristics and work information of the study subjects are described using mean ± standard deviation for continuous variables and frequency for categorical variables.

### Influence factor analysis

#### Analysis based on preferences for LTCI compensation strategies from DCEs

Taking the preference choices of LTCI respondents as the dependent variable, and care content, per service cost, care residents, frequency of care, and compensation method as independent variables, a conditional Logit model was established. In this model, the first level of each attribute was set as the base level, and regression coefficients were used to indicate the degree of preference for other levels relative to the first level. The theoretical foundation of the analysis of DCEs is the random utility theory. Utility in economics refers to the satisfaction consumers derive from consuming a certain good or service. The theory assumes that an individual *n* will choose the policy option with the highest utility (*U*) from *J* alternative policy options, i.e. the individual will choose policy option *i* over policy option *j* only when *Uni* > *Unj*. The utility *U* can be expressed as:

*Uni* = *Vni* + ε*ni* = β_1l_Living care + β_2_Medical care + β_3_Family + β_4_Registered nurse + β_5_Caregiver + β_6_Per service cost + β_7_Mixed compensation + β_8_Cash compensation + β_9_Proportional reimbursement + ε*ni* (1) where *Vni* is the observable part of the model, and ε*ni* is the unobservable part, reflecting uncertainty factors such as random errors and individual variability in preferences.

When two policy options are presented to individual *n* simultaneously, the probability (*Pni*) of choosing policy option i over policy option *j* can be expressed as


(2) $$\begin{aligned}{\mathrm{P(ni}}\mid \left( {{\mathrm{i,j}}} \right){\mathrm{) = Pr}}\left[ {{\mathrm{Uni > Unj}}} \right] \cdot {\mathrm{i = j}} {{\epsilon} {J}}\end{aligned}$$

Combining this with equation (1), we have:


(3) $$\begin{aligned}{\mathrm{P(ni}}\mid \left( {{\mathrm{i,j}}} \right){\mathrm{) = Pr}} & \left[ {{\mathrm{Vni}}} {+ \varepsilon {\mathrm{ni}} > Vnj + \varepsilon nj} \right]{\mathrm{ = Pr}}\left[ {{\varepsilon nj - \varepsilon}} \right. \\ & \nonumber \left. {{ni < Vni - Vnj}} \right] \cdot {\mathrm{i = j}} {{\epsilon J}}\end{aligned}$$

In the analysis of discrete choice experiments, assuming ε*nj* − ε*ni* follows a logistic distribution, according to probability theory, the probability of all subjects choosing institution *i* can be derived as equation (3). The coefficients in equation (1) can be estimated using the maximum likelihood method, and this step can be implemented using STATA’s clogit module.

#### Estimation of willingness-to-pay in this study

‘Per service cost’ was treated as a continuous variable for estimation. The willingness-to-pay for other characteristic factors was estimated by calculating the negative ratio of the regression coefficients of other features to the cost coefficient. For instance, the willingness-to-pay for care personnel was calculated by ‘−β_Family/β_Per service cost’, indicating the average additional cost respondents are willing to pay for a policy option with family as the care personnel.

Moreover, by substituting the coefficient values of the respective feature levels in equation (3), the impact of changing the level of a certain feature factor on the choice can be calculated. For example, when other feature factor levels are at the baseline level, changing the care personnel from caregiver to family means the change in choice probability is P_Family_− P_Caregiver_. Finally, subgroup analyses were conducted for subjects of different genders, educational levels, different ADL ratings, and different daily caregivers, to compare the preferences for LTCI policy choices of residents with different background characteristics.

## Ethical approval

The study has been approved by the Biomedical Ethics Committee of Southern Medical University (NFYKDX001). An informed consent waiver has been applied for this study.

## Results

### Basic information of respondents

This study initially identified a total of 156 individuals receiving LTCI services in the selected community. Among them, 27 individuals were excluded due to severe illness that prevented normal communication, 9 individuals were excluded due to mental health conditions, and 24 individuals declined participation citing a lack of understanding of the study. Ultimately, 96 eligible participants were included in the study. These participants represent a diverse range of backgrounds and characteristics, which are detailed below.

In this study, a total of 96 samples were collected, with males and females constituting 41.6% and 58.4%, respectively. The average age of all respondents was 76.0 years. Approximately 48.9% of the participants had received education at the secondary level or above. More than half of the participants (55.2%) had a family income in the range of 3001–5999 CNY. The marital status was predominantly married and cohabiting, accounting for 70.8%. The majority of the respondents had local household registration, making up 86.5%, of whom 52.1% participated in urban and rural resident medical insurance, and 47.9% were covered by urban employee medical insurance. The occupational backgrounds prior to retirement included employees of institutions, company employees, self-employed individuals, farmers, and others, comprising 19.8%, 28.1%, 10.4%, 21.9%, and 19.8%, respectively. At the time of the survey, 77.1% of the respondents were assessed as disabled through ADL evaluation, with 40.6% being severely disabled. Some respondents (22.9%) had recovered to a normal ADL level after receiving long-term care. Nearly half of the respondents lived with their spouse or with their spouse’s children, with spouses being the primary daily caregivers, accounting for 56.3%. The vast majority of respondents had good relationships with their family members, with as high as 95.8% of the respondents rating their family members as very caring. Details are shown in Table 2.

**Table 2.** Descriptive analysis

Variable	Composition ratio [*n* (%)]	Variable	Composition ratio [*n* (%)]
Gender		**Marital status**	
Male	40 (41.6)	Single (unmarried, divorced, separated, widowed, etc.)	28 (29.2)
Female	56 (58.4)	Married (married and living together)	68 (70.8)
**Age^a^ (years)**	76.0 ± 11.8	**Basic medical insurance**	
**Education level**		Medical insurance for urban and rural residents	50 (52.1)
Primary and below	50 (52.1)	Urban and rural workers' medical insurance	46 (47.9)
Secondary and above	46 (48.9)	**Household Type**	
Household registration		Non-agricultural household	33 (34.4)
Local household	83 (86.5)	Agricultural household	63 (65.6)
Non-local household registration	13 (13.5)	**Occupation before retirement**	
**Competency assessment level**		Employee of public organization	19 (19.8)
Normal^b^	22 (22.9)	Corporate employee	27 (28.1)
Mild disability	17 (17.7)	Self-employed	10 (10.4)
Moderate disability	18 (18.8)	Farmer	21 (21.9)
Severe disability	39 (40.6)	Other	19 (19.8)
**Living conditions**		**Daily caregiver**	
Living with spouse only	34 (35.4)	Irregular caregiver	20 (20.8)
Living with spouse and children	43 (44.8)	Spouse	54 (56.3)
Other	19 (19.8)	Paid caregiver (nanny, caregiver, etc.)	22 (22.9)
**Average monthly household income (CNY)**		**Family's level of concern**	
≤3000	17 (17.7)	Very concerned	92 (95.8)
3001–5999	53 (55.2)	Generally concerned	3 (3.1)
≥6000	26 (27.1)	Not concerned	1 (1.1)

a Mean ± standard deviation.

b Back to normal at the time of the survey.

### Analysis of policy preferences among respondents

The model results indicate that, among the five attributes included in the study, all but the frequency of care significantly influenced the respondents’ preferences for LTCI choices (*P* < 0.05). Concerning care content, with living care as the baseline, the coefficient value for medical care was β = 0.527, indicating that residents prefer care content primarily focused on medical care. In terms of per service cost, residents showed a preference for lower-priced options (β = −0.003). Regarding care personnel, with family members as the baseline, the coefficient values for registered nurses and caregivers were β = −0.467 and β = −0.701, respectively, indicating that respondents prefer family members as their care personnel. As for the compensation method, residents showed a preference for cash subsidies (β = 0.618). Details are shown in Table 3.

**Table 3.** Analysis of discrete selection test results

		Total number of observations: 1428			
Characteristic factors	Level	Coefficient	SE	CI^a^ (2.5%)	CI (97.5%)
**Content of care**	Life care is the mainstay	–	–	–	–
	Medical care	0.527**	0.149	0.235	0.820
**Average per capita cost**	–	−0.003***	0.001	−0.005	−0.001
**Nursing staff**	Family members	–	–	–	–
	Registered nurse	−0.467 *	0.202	−0.864	−0.071
	Care worker	−0.701**	0.102	−1.096	−0.305
**Frequency of care**	4 h 7 times/week	–	–	–	–
	1 h 7 times/week	0.070	0.204	−0.330	0.470
	2 h 1 time/week	−0.135	0.206	−0.539	0.268
**Mode of compensation**	Mixed benefits	–	–	–	–
	Proportional reimbursement	0.206	0.210	−0.205	0.617
	Cash subsidy	0.618***	0.207	0.212	1.024
const**^b^**	–	0.123	0.412	−0.171	0.417
**Log likelihood**		−457.606			

* *P* < 0.05,

** *P* < 0.01,

*** *P* < 0.001.

^a^Confidence interval.

^b^const: Intercept term in the conditional logit model, representing the baseline choice effect when all other variables are at their reference levels.

### Willingness-to-pay for policy preferences among respondents

The results indicate that in terms of care content, compared to living care, residents are willing to pay an additional 157.92 yuan to receive medical care. Regarding care personnel, if respondents receive a compensation of 139.90 yuan, they are willing to switch their care personnel from family members to registered nurses. If residents receive a compensation of 209.79 yuan, they are willing to change their care personnel from family members to caregivers. In terms of compensation method, compared to mixed compensation, residents are willing to pay an extra 184.94 yuan to receive compensation in the form of cash subsidies. As for the frequency of care, the study results show no difference in the willingness-to-pay for different care frequencies. Details are shown in Table 4.

**Table 4.** Analysis of willingness-to-pay results

	Willingness-to-pay	Minimum CI^a^ (2.5%)	Maximum CI (97.5%)
**Content of care**			
Life care is the mainstay	–	–	–
Medical care	157.92*	25.80	290.03
**Nursing staff**			
Family members	–	–	–
Registered nurse	−139.90*	−250.92	−28.89
Care worker	−209.79*	−381.63	−37.95
**Frequency of care**			
4 h 7 times/week	–	–	–
1 h 7 times/week	20.90	−95.59	137.40
2 h Once/week	−40.47	−169.84	88.89
**Mode of compensation**			
Mixed benefits	–	–	–
Proportional reimbursement	61.63	−81.92	205.18
Cash subsidy	184.94*	3.88	366.00

* P < 0.05

** P < 0.01

*** *P* < 0.001.

a Confidence interval.

### Differences in preferences for LTCI services among respondents with different characteristics

To further analyze the impact of population heterogeneity on compensation preferences, subgroup analyses were conducted from four aspects: gender, education, ADL rating, and caregivers. The gender subgroup analysis model results showed that the attributes of per service cost and care personnel had an impact on policy preferences for male respondents. Additionally, the model results indicated that the attribute of compensation method affected policy preferences for female respondents, with female respondents preferring cash subsidies over mixed compensation (β = 0.836). The educational level subgroup analysis model results revealed that the attributes of care content, per service cost, and care personnel influenced policy preferences for respondents with secondary education or above. Furthermore, the model results showed that the attribute of compensation method influenced policy preferences for respondents with less than secondary education, with these respondents preferring cash subsidies over mixed compensation (β = 0.644). The ADL rating subgroup analysis model results indicated that per service cost and care personnel attributes influenced policy preferences for respondents with normal ADL ratings. Moreover, the model results showed that the attribute of care content influenced policy preferences for respondents with mild to moderate disability and severe disability in ADL ratings. Relative to living care, respondents with mild to moderate disability (β = 0.619) and severe disability (β = 0.479) both preferred medical care. Besides care content, the attributes of care personnel and compensation method also influenced policy preferences for respondents with severe disability in ADL ratings. The daily caregiver subgroup analysis model results showed that the attribute of per service cost influenced policy preferences for respondents with a nanny or children/spouse as caregivers, with both types of respondents preferring lower-priced costs, indicating a desire to spend less on LTCI services, with coefficients values of β = 0.026 and β = 0.018, respectively. Additionally, the attributes of care content, care personnel, and compensation method influenced policy preferences for respondents with children or family members as caregivers and those with no fixed or other caregivers. (Details are given in supplementary Tables S3–S6 in the online supplementary material.)

## Discussion

This study utilized the DCE method to systematically explore the preferences and willingness-to-pay of home-based disabled elderly individuals in Guangzhou for LTCI compensation mechanisms, while analyzing the influence of demographic characteristics on these preferences. This research not only provides critical empirical support for optimizing LTCI policies in China but also offers valuable insights for designing LTCI policies for elderly populations in low- and middle-income countries (LMICs) globally. Compared to existing studies, this study focuses on home-based care as the primary service model, refining the measurement of preferences among disabled elderly populations and addressing gaps in the analysis of compensation mechanisms and preference heterogeneity.

The study results indicate that respondents showed a stronger preference for medical care over life care. This finding aligns with the growing demand for medical services among the elderly population in China in recent years (Akobirshoev et al. 2022, Aparicio Betancourt et al. 2022). With the increasing prevalence of chronic diseases that is intensified in an aging population, the unequal distribution of medical resources has made disabled elderly individuals more dependent on professional medical care. This finding also supports the theory of Grossman’s health demand model, which posits that deteriorating health conditions lead to increased service demand (Grossman 1972). Policymakers should focus on expanding the coverage of medical care services, particularly by enhancing resource investment in primary healthcare institutions and community care centers, to ensure the equity and accessibility of medical services and meet the actual needs of disabled elderly populations.

Furthermore, this study found that having family members as caregivers is a significant preference among respondents, aligning closely with the values of traditional family-based elder care in Chinese culture (Förster et al. 2019). Family caregiving is not only a cultural tradition but also a crucial component of long-term care services. However, with the family structure changes brought about by the ‘one-child policy’ the ‘4-2-1’, family model has led to an increasing shortage of family caregiving resources (Gustafson and Baofeng 2014). The findings underscore the need for future policy designs to address these challenges by introducing professional caregivers to supplement family caregiving. At the same time, skill training and financial subsidies should be provided to support family caregivers. Integrating family caregiving with professional services into a comprehensive care model can help alleviate caregiving pressures and improve service quality.

This study also found that respondents generally preferred cash subsidies as a compensation method. This preference may stem from the simplicity and flexibility of cash subsidies, enhancing the appeal of LTCI policies (Dou et al. 2018). Female respondents showed a particularly strong preference for cash subsidies, which might be associated with their leading role in household financial management (Central University of Finance and Economics 2021). Additionally, the results of the subgroup analysis showed that individuals with severe disabilities preferred cash subsidies, a seemingly counterintuitive finding (as it is typically assumed that high-need groups are more inclined towards proportional reimbursement). This highlights the need for more refined policy design. A possible explanation is that severely disabled individuals, due to the long-term and high-frequency nature of their care needs, are more concerned with the ‘predictability’ of reimbursement amounts (Shen et al. 2014). In contrast, the moderate to mild disability group may have intermittent care needs, making proportional reimbursement a more flexible option to match their fluctuating expenditures. This suggests that policymakers could implement a cap system for high-frequency care to manage overall expenditure, while adopting a proportional system for low-frequency care to offer greater flexibility in fund utilization. Such a dual approach would prevent overspending and fund stagnation, ensuring a balance between efficiency and sustainability. Future policy designs should explore flexible compensation models that integrate both cash subsidies and proportional reimbursement, fostering long-term policy stability while accommodating diverse care needs.

In terms of willingness-to-pay, the study revealed significant heterogeneity. Respondents with higher education levels were more likely to prefer medical care, while those with lower education levels prioritized lower-cost services. This indicates that education plays an important role in shaping health cognition and preferences (Lehnert et al. 2018, He et al. 2021). Additionally, respondents with higher ADL scores (i.e. the severely disabled population) exhibited a stronger reliance on professional caregivers, showing a preference for registered nurses and professional care providers. Designing differentiated compensation strategies for these specific groups can help enhance the inclusiveness and precision of policies.

Moreover, the results of this study demonstrate both consistency and significant differences when compared to other domestic LTCI studies using the DCE method. For instance, existing studies have generally highlighted that the choice of care content and compensation methods among elderly residents is influenced by multiple factors (Wang et al. 2021b, Ma et al. 2023a). This study further focuses on home-based disabled elderly individuals, confirming the significance of medical care and family care as key attributes, complementing prior research on preferences among a broader elderly population (Ma et al. 2023b, Jiang et al. 2024). Additionally, this study found that respondents showed a stronger preference for cash subsidies, aligning with the convenience demands in policy implementation and consistent with the emphasis on flexible compensation mechanisms in some studies (Dou et al. 2018).

This study also reveals significant heterogeneity in respondents’ willingness-to-pay, particularly in terms of the influence of education level and ADL scores on their choice behaviors. This is consistent with previous research exploring the impact of demographic characteristics on preferences (Chandoevwit and Wasi 2020, Wang et al. 2022b) (Chen et al. 2020). In contrast to some studies that focus on a broader set of attributes (such as waiting time or service accessibility), this study highlights five core attributes and refines the understanding of individual preferences through scenario simulations.

### Strengths and limitations

This study’s primary contribution lies in quantifying the preferences and willingness-to-pay of home-based disabled elderly individuals for LTCI services using the DCE method, providing critical data support for optimizing compensation mechanisms. The findings reveal heterogeneous preferences and offer practical recommendations for policy optimization, while also demonstrating the potential of DCE for exploring health policy preferences in the context of LMICs.

However, the study has certain limitations. First, the sample was restricted to Guangzhou, which may limit the generalizability of the findings. Future research could expand to other regions to verify its broader applicability. Second, the hypothetical scenarios in the experimental design may not fully reflect real-life situations. Future studies could incorporate actual service data to enhance external validity. Additionally, this study focused on five core attributes, leaving out other relevant factors such as waiting times or service accessibility. Future research could broaden the scope of analysis to gain a more comprehensive understanding of LTCI preference characteristics.

## Conclusion

This study provides critical empirical evidence for optimizing China’s LTCI policy and offers valuable insights for designing LTCI systems targeting the elderly population in LMICs. The findings underscore the importance of expanding the coverage of medical care services and suggest strengthening resource investment in primary healthcare institutions and community care centers to enhance service equity and accessibility.

In supporting family caregivers, policy design should focus on alleviating caregiving pressures through skills training and financial subsidies, while introducing professional caregivers to establish an integrated care model that combines family-based care with professional services.

Given the significant preference for cash subsidies among respondents, policies should explore flexible and diverse compensation schemes that combine cash subsidies with proportional reimbursement models. This approach would balance flexibility and financial sustainability to better meet diverse needs. Moreover, policy design must account for demographic heterogeneity by formulating differentiated compensation strategies tailored to groups with varying education levels, genders, and ADL scores, thereby enhancing inclusivity and precision. Through multi-layered and comprehensive policy optimization, China’s LTCI system is expected to better address the challenges of population aging and serve as a reference and inspiration for other nations.

## Supplementary Material

czaf015_Supp

## Reflexivity statement

The authors represent a range of gender identities, locations, and seniority. Authors are from China which is classified as a lower-middle-income country. The authors include nine women and two men, spanning multiple levels of experience. Among them, two authors specialize in Chinese health policy and management, two are practitioners at grassroots health units in China, i.e. unit staff, one is a graduate student majoring in health policy and management, and the remaining six authors are undergraduates with majors covering health economics, health law, and nursing. All authors have extensive experience in conducting qualitative field studies in Guangzhou, China, especially in community health centers and the primary healthcare sector in Guangzhou.

## Abbreviations

ADL, Activities of daily living; DCE, Discrete choice experiment; LMICs: Low- and middle-income countries; LTCI, Long-term care insurance.
