# Supplementary material for: Compensation preferences of home-based disabled beneficiaries in the long-term care insurance system in Guangzhou, China
Source: Health Policy Plan. 2025 Apr 1;40(5):556–65. doi: 10.1093/heapol/czaf015 (PMC12063582; doi:10.1093/heapol/czaf015)
Supplement: czaf015_Supp [file czaf015_supp.zip › suppl_data/Appendix.docx]

**Appendix 1**

Introduction to the Survey Questionnaire

Dear Participant,

Thank you very much for taking the time to participate in this survey. To better design new long-term care insurance (LTCI) schemes, we aim to understand your genuine needs and preferences. Your feedback will help us create compensation plans that better align with your actual requirements.

In the following questionnaire, you will encounter several choice-based questions regarding different LTCI schemes. Each question presents two options (schemes) for your comparison and selection. Each scheme is composed of five aspects (referred to as “attributes”), which are combined at different levels. The definitions of the attributes and levels included in the questionnaire are as follows:

1. Content of Care

Content of care refers to the primary types of services provided in long-term care. Based on service goals and your needs, the content of care can be divided into two categories: medical care and life care.

1. Medical care focuses on health management and medical support, aiming to help beneficiaries maintain or improve their health and prevent deterioration. Specific services include health monitoring, such as measuring blood pressure, blood sugar, and body temperature; rehabilitation therapy, including physical therapy and functional training; wound care, such as dressing changes and debridement; and medication management, assisting beneficiaries in taking medications on time or adjusting medication plans. Medical care is more suitable for beneficiaries with specific medical needs or chronic disease management requirements, such as individuals with diabetes, heart disease, or other conditions requiring regular health monitoring and intervention.

(2) Life care primarily refers to non-medical daily living support and assistance, aiming to help beneficiaries complete daily activities and improve their quality of life. Specific services include assisting with daily activities such as dressing, bathing, and eating; providing household support such as cleaning, laundry, and cooking; companionship services including chatting and offering psychological comfort to reduce loneliness; and outing support, such as assisting with shopping and accompanying medical visits. Life care is more suitable for beneficiaries who require additional assistance in daily living but are otherwise physically stable, such as those with mobility issues or basic daily care needs.

**Filling Tips:** When answering the questionnaire, you can choose the type of care that best suits your needs. For example, if you value daily living support, such as help with household chores or outings, you may prefer life care. If you require more health management or rehabilitation services, such as dressing changes or regular health monitoring, you may prefer medical care.

1. Average per capita cost

Average per capita cost refers to the range of out-of-pocket expenses required for each care service (i.e., per-service cost). This cost directly affects the affordability of individual care services for respondents.

1. Out-of-pocket less than 100 yuan: Each care service requires a relatively low out-of-pocket cost, totaling less than 100 yuan. This represents a lighter financial burden, making it suitable for families or individuals with limited budgets. Services under this cost range may include basic care options, such as short-duration life care or low-frequency medical support.
2. Out-of-pocket 100-300 yuan: Each care service requires an out-of-pocket cost between 100 and 300 yuan. This represents a moderate financial burden and allows for access to more diverse care services. Services under this range may include a combination of life care and medical care or care services of longer duration.
3. Out-of-pocket payment of more than 300 yuan: Each care service requires a relatively high out-of-pocket cost, totaling more than 300 yuan. This represents a heavier financial burden, making it suitable for beneficiaries with greater care needs and stronger economic capabilities. Services under this cost range may include high-quality or complex medical care provided by professional caregivers.

**Filling Tips:** When answering the questionnaire, respondents should select the option that best matches their financial capacity and individual care service needs. For example, if you prioritize saving costs or require simpler services, you may prefer “out-of-pocket less than 100 yuan per service.” If you seek more comprehensive or higher-quality care services and can accept higher costs, you may prefer “out-of-pocket payment of more than 300 yuan per service.”

1. Nursing Staff

Nursing staff refers to the individuals providing direct care services to beneficiaries in long-term care. Depending on their identity and professional skills, the content and quality of care they deliver may vary.

1. Family Members: Family members serve as the primary caregivers, providing care services to beneficiaries. Family caregivers may include spouses, children, or other relatives. Their services typically rely on non-professional skills and daily life experience, focusing primarily on life care, such as assisting with bathing, cooking, or daily companionship.
2. Care Worker: Care workers are professionally trained personnel who provide care services to beneficiaries. They possess basic caregiving skills, enabling them to deliver life care and some simple medical care. Care workers are typically employed by community care institutions or private caregiving companies. They are more professional than family caregivers but have limited capacity for medical care.
3. Registered Nurse: Registered nurses are professionally trained and certified medical personnel. They can provide high-level medical care services, such as health monitoring, wound dressing, and rehabilitation guidance. Registered nurses are often affiliated with medical institutions or advanced caregiving teams. Their services are more systematic and professional, meeting complex medical care needs. They are suitable for beneficiaries requiring ongoing health monitoring or intensive medical care, such as chronic disease patients or individuals recovering from surgery.

**Filling Tips:** When answering the questionnaire, respondents should select the type of caregiver that best meets their needs and level of trust. For example, if you prefer a familiar family environment and have lower care needs, you may opt for “Family Members.” If you seek a moderate level of professional care at a reasonable cost, you may choose “Care Worker.” If you have significant medical needs or trust professional medical care more, you may prefer “Registered Nurse.”

1. Frequency of Care：

Frequency of care refers to the scheduling of care services, including the number of care sessions per week and the duration of each session. Care frequency affects the overall coverage and intensity of care and should be selected based on the specific needs of the beneficiary.

(1) 4 hours * 7 times/week: Care is provided 7 times a week, with each session lasting 4 hours. This arrangement offers high-frequency and long-duration care, suitable for beneficiaries requiring continuous daily support. It can meet comprehensive needs for life care and some medical care, such as daily assistance with living activities and rehabilitation training. This is ideal for beneficiaries with high dependency, such as those with limited mobility or requiring long-term monitoring.

(2) 1 hour * 7 times/week: Care is provided 7 times a week, with each session lasting 1 hour. This arrangement offers high-frequency but shorter-duration care, suitable for beneficiaries needing regular monitoring or brief support. It is often focused on medical care, such as daily health monitoring or medication management. This is appropriate for beneficiaries with stable health conditions who need small-scale daily support.

(3) 2 hours * 1 time/week: Care is provided once a week, with each session lasting 2 hours. This arrangement offers low-frequency but concentrated-duration care, suitable for beneficiaries with relatively fewer needs. It often focuses on specific tasks, such as periodic rehabilitation guidance or comprehensive health evaluations. This is ideal for those with limited budgets or low care needs.

**Filling Tips:** When answering the questionnaire, respondents should choose the care frequency that best matches their needs and budget. For example, if you require continuous daily support, you may prefer “4 hours * 7 times/week.” If your needs lean toward regular monitoring and shorter care sessions, you may prefer “1 hour * 7 times/week.” If you have fewer needs or only require focused care, you may opt for “2 hours * 1 time/week.”

1. Mode of Compensation

Mode of compensation refers to the specific forms of financial support provided to beneficiaries under the long-term care insurance (LTCI) system. This directly affects how beneficiaries receive reimbursement and the flexibility of the compensation.

1. Mixed benefits: This mode combines cash subsidies and proportional reimbursement, offering flexible and diversified financial support. Beneficiaries can receive a fixed cash subsidy to cover daily expenses and also reduce high costs through proportional reimbursement. This mode is suitable for beneficiaries with diverse care needs, requiring both fixed support and additional reimbursement. It is particularly appropriate for families with complex care requirements and varying financial capacities.
2. Proportional reimbursement: This mode reimburses care costs based on a fixed percentage of the beneficiary’s actual expenses. The amount reimbursed is directly linked to the actual expenditure. For example, if a beneficiary incurs costs of 300 yuan, a 50% reimbursement rate would provide 150 yuan. This mode is better suited for beneficiaries requiring higher amounts or more frequent care services, especially those with significant medical care needs.
3. Cash subsidy: This mode provides a fixed amount of subsidy per month, regardless of the actual cost of care services. For example, beneficiaries might receive a fixed 80 yuan subsidy each month, regardless of their actual care expenses. This mode provides stable financial support, making it more suitable for beneficiaries with limited budgets or irregular care needs. It is ideal for those needing consistent financial aid but with relatively low care service expenditures.

**Filling Tips:** When answering the questionnaire, respondents should choose the mode of compensation that best matches their care cost needs and preferences. For example, if you need stable financial support to cover daily expenses and have low care needs, you may prefer cash subsidy. If you want flexible reimbursement based on actual expenses and have higher care service costs, you may prefer proportional reimbursement. If you need a combination of fixed subsidies and proportional reimbursement to address diverse needs, you may prefer mixed benefits.

**eTable. Attributes and Levels**

| **Characteristic factors** | **Level** | | |
| --- | --- | --- | --- |
| **Content of care** | Life care is the mainstay | Medical care |  |
| **Average per capita cost** | Out-of-pocket less than 100 yuan | Out-of-pocket 100-300 yuan | Out-of-pocket payment of more than 300 yuan |
| **Nursing staff** | Family members | Care Worker | Registered Nurse |
| **Frequency of care** | 4 hours*7 times/week | 1 hour * 7 times / week | 2 hours * 1 time / week |
| **Mode of compensation** | Mixed benefits | Proportional reimbursement | Cash subsidy |

**Appendix 2 This Questionnaire’s Purpose and Instructions**

This questionnaire is designed to understand your preferences for different long-term care insurance (LTCI) schemes. To help you complete the questionnaire smoothly, the following instructions and guidelines are provided.

**Content of the Questionnaire**

1. Each question presents two different care schemes, which are composed of different combinations of five attributes:

• **Content of care**: Medical care or life care.

• **Per-service cost**: Costs per care session fall into three ranges (<100 yuan, 100-300 yuan, >300 yuan).

• **Nursing staff**: Family members, care workers, or registered nurses.

• **Frequency of care**: Different combinations of weekly care frequency and duration.

• **Mode of compensation:** Cash subsidy, proportional reimbursement, or mixed benefits.

**2. Compare the two schemes**

Each scheme will display different levels of these five attributes. Your task is to compare the two schemes and select the one that best matches your actual needs and preferences.

**Steps for Completing the Questionnaire**

**1. Carefully read the details of the two schemes**

• Compare the levels of the five attributes, such as content of care, cost, nursing staff, frequency of care, and mode of compensation.

• Consider whether each scheme aligns with your personal needs and preferences.

**2. Select the scheme that best suits you**

• Based on your living conditions and financial capacity, choose the scheme that is more suitable for you.

• There are no “right” or “wrong” answers; simply select the scheme that you believe will benefit you the most.

**3. Complete all questions**

• For each question, you must choose one scheme out of the two presented. Please read the descriptions carefully before making your selection.

**Sample Question**

Here is an example to help you understand how to complete the questionnaire:

**Question: Below are two LTCI schemes. Please choose the one you prefer.**

| Attribute | Scheme 1 | Scheme 2 |
| --- | --- | --- |
| Content of care | Life care | Medical care |
| Per-service cost | Out-of-pocket less than 100 yuan | Out-of-pocket 100-300 yuan |
| Nursing staff | Family members | Registered nurse |
| Frequency of care | 4 hours * 7 times/week | 2 hours * 1 time/week |
| Mode of compensation | Cash subsidy | Proportional reimbursement |
| Which type of benefits would you prefer? | **□** | **□** |

Your Choice:

• If you value a home-like caregiving environment, lower costs, flexible caregiving frequency, and prefer a cash subsidy for compensation, you may lean towards choosing Scheme 1.

• If you require professional medical care, are willing to accept higher out-of-pocket costs, and prefer proportional reimbursement for compensation, while accepting less frequent but professional caregiving, you may opt for Scheme 2.

**Filling Tips:**

• Express your true preferences: Please make your choices based on your actual care needs and preferences. There is no need to align with any specific answer.

• Choose for each question: Compare the two schemes in each question and make your selection.

Thank you for your patience in completing this questionnaire. Your responses will provide valuable insights for improving long-term care insurance schemes!

**Appendix 3 Guangzhou Long-Term Care Insurance (LTCI) Trial Implementation Measures**

**Chapter 1: General Provisions**

Article 1

To improve the social security system and meet the basic care needs of disabled individuals in Guangzhou, these measures are formulated in accordance with the “Guiding Opinions on Expanding the Long-Term Care Insurance System Pilot” (Medical Insurance [2020] No. 37) issued by the National Healthcare Security Administration and the Ministry of Finance, combined with the actual conditions in Guangzhou.

Article 2

These measures apply to activities related to LTCI enrollment, funding, benefit utilization, management, and operations within the administrative region of Guangzhou.

Article 3

The Guangzhou Healthcare Security Administration is responsible for LTCI management and policy implementation. Its duties include:

1. Developing LTCI policies and overseeing their implementation.

2. Guiding LTCI operational management.

3. Supervising policy execution.

The Guangzhou Medical Insurance Agency (hereinafter referred to as the “Medical Insurance Agency”) is responsible for LTCI enrollment, funding, operational services, and agreement management. Other relevant departments, including finance, civil affairs, health, and human resources, shall carry out related tasks within their respective responsibilities.

**Chapter 2: Enrollment and Contributions**

Article 4

All participants in the city’s employee and urban-rural resident medical insurance programs aged 18 and above are automatically enrolled in LTCI. The Medical Insurance Agency will establish LTCI enrollment records for them.

Article 5

LTCI is funded through contributions from employers and individuals, as well as government subsidies:

1. For employees, contributions consist of deductions from employer and personal accounts.

2. For residents, contributions include personal payments and fiscal subsidies.

Article 6

The contribution base and rates are as follows:

1. For employees:

• The contribution base is the annual employee medical insurance contribution base, with monthly rates varying by age and employment status.

• Individuals aged under 35 are exempt from personal contributions; those aged 35 and above contribute between 0.02% and 0.12%.

2. For residents:

• Annual rates for individuals aged 18 and above are set at 0.12%, split equally between personal contributions and fiscal subsidies.

**Chapter 3: Benefits and Payments**

Article 7

LTCI funds cover the following costs:

1. Bed fees at designated care institutions.

2. Fees for living assistance, medical care, and associated consumables.

3. Assessment fees for eligibility evaluations.

Article 8

The following costs are not covered by LTCI funds:

1. Expenses already covered by other insurance programs (e.g., medical, work injury, or maternity insurance).

2. Costs exceeding LTCI coverage standards or incurred at non-designated facilities.

Article 9

Eligibility assessments are required for LTCI benefits, including:

1. Disability assessments.

2. Extended care assessments.

3. Equipment usage assessments.

Article 10

Beneficiaries must meet one of the following conditions to qualify for LTCI benefits:

1. Long-term disability lasting six months or more, with stable medical conditions assessed as levels 1-3.

2. Discharged hospital patients with specific post-treatment care needs.

**Chapter 4: Management**

Article 20

The Medical Insurance Agency is responsible for managing LTCI operations, including enrollment, fund allocation, and benefit approval.

Article 21

Institutions providing LTCI services must meet qualification requirements and sign service agreements with the Medical Insurance Agency.

Chapter 5: Fund Management

Article 25

LTCI funds come from:

1. Transfers from employee medical insurance funds.

2. Individual contributions and government subsidies.

3. Interest income and other legitimate sources.

Article 26

LTCI funds are managed under separate accounts for employees and residents and are strictly allocated for LTCI purposes.

Article 27

Financial management and accounting of LTCI funds are carried out in accordance with national regulations.

The above content is sourced from the Guangzhou Administrative Normative Document Platform: Notice on the Issuance of the Guangzhou Long-Term Care Insurance Trial Implementation Measures by the Guangzhou Healthcare Security Administration, Guangzhou Finance Bureau, Guangzhou Civil Affairs Bureau, and Guangzhou Health Commission (https://www.gz.gov.cn/gfxwj/sbmgfxwj/gzsylbzj/content/post_7005791.html?utm_source=chatgpt.com).
