# Supplementary material for: Compensation preferences of home-based disabled beneficiaries in the long-term care insurance system in Guangzhou, China
Source: Health Policy Plan. 2025 Apr 1;40(5):556–65. doi: 10.1093/heapol/czaf015 (PMC12063582; doi:10.1093/heapol/czaf015)
Supplement: czaf015_Supp [file czaf015_supp.zip › suppl_data/Supplementary Table 1 Categorization and Definitions of Attributes in the Study.docx]

**Supplementary Table 1 Categorization and Definitions of Attributes in the Study**

| **dimensions** | **Characteristic factors** | **Definition** |
| --- | --- | --- |
| **Service Features** | Care Content | Refers to the specific type of service provided, such as daily living support (e.g., bathing, dressing, eating) or medical care (e.g., wound dressing, medication management, rehabilitation). |
|  | Frequency of Care | Refers to how often care is provided, such as daily, weekly, or multiple times per day. |
|  | Service Period | Refers to the total duration for which the service is provided, such as one month, six months, or over a year. |
|  | Service Time | Refers to the specific timing of the service, such as morning, afternoon, or full-day sessions. |
| **Service Delivery** | Care Personnel | Refers to the individuals delivering the care, including family members, registered nurses, or caregivers trained in basic care. |
|  | Service Location | Refers to where the care services are provided, such as at the beneficiary’s home, a community health center, or a dedicated care institution. |
|  | Service Institution | Refers to the organizations or entities providing the service, such as public health institutions, private care agencies, or community-based organizations. |
|  | Reservation Method | Refers to how beneficiaries can book services, such as via phone, online platforms, or in-person appointments at service centers. |
| **Economic Support** | Per Service Cost | Refers to the cost of each service session, which may vary based on the type of care and service provider. |
|  | Compensation Method | Refers to the economic mechanism for reimbursing the costs of long-term care services, which can vary based on the approach used, such as fixed daily compensation, proportional reimbursement, or a mixed model combining both. |

**Supplementary Table 2 Frequency Statistics of Experts’ Selection of Long-Term Care Insurance Attributes**

| **Attribute** | **Frequency of Selection** | **Notes** |
| --- | --- | --- |
| Care Content | 12 | Frequently mentioned in both literature and expert feedback. |
| Per Service Cost | 5 | Highlighted as a key economic factor. |
| Care Personnel | 3 | Relevant to service delivery quality. |
| Frequency of Care | 3 | Reflects intensity of care needs. |
| Compensation Method | 3 | Influences user affordability and preferences. |
| Service Period | 2 | Considered less critical by most experts. |
| Service Location | 2 | Important but secondary to other attributes. |
| Service Institution | 1 | Added based on expert recommendations. |
| Reservation Method | 0 | Not prioritized by experts. |
| Service Time | 0 | Considered non-essential. |

**Supplementary Table 3 Discrete Choice Experiment Gender Subgroup Results Analysis.**

| **Characteristic factors** | **Level** | **male** | | | **female** | | |
| --- | --- | --- | --- | --- | --- | --- | --- |
|  |  | **β** | **P** | **95%CI** | **β** | **P** | **95%CI** |
| **Content of care** | Life care is the mainstay | – | – | – | – | – | – |
|  | Medical care | 0.672 | 0.002 | (0.244,1.100) | 0.419 | 0.045 | (0.009,0.829) |
| **Average per capita cost** | – | -0.006 | ＜0.001 | (-0.009,0.003) | -0.001 | 0.601 | (-0.004,0.002) |
| **Nursing staff** | Family members | – | – | – | – | – | – |
|  | Registered Nurse | -0.818 | 0.006 | (-1.402,-0.233) | -0.135 | 0.632 | (-0.686,0.417) |
|  | Care Worker | -0.969 | 0.001 | (-1.543,-0.395) | -0.444 | 0.119 | (-1.003,0.114) |
| **Frequency of care** | 4 hours*7 times/week | – | – | – | – | – | – |
|  | 1 hour * 7 times / week | 0.212 | 0.466 | (-0.357,0.781) | -0.096 | 0.742 | (-0.667,0.475) |
|  | 2 hours * 1 time / week | -0.115 | 0.697 | (-0.690,0.462) | -0.144 | 0.623 | (-0.715,0.428) |
| **Mode of compensation** | Mixed benefits | – | – | – | – | – | – |
|  | Proportional reimbursement | 0.003 | 0.993 | (-0.586,0.591) | 0.414 | 0.166 | (-0.171,1.000) |
|  | Cash subsidy | 0.423 | 0.149 | (-0.152,0.998) | 0.836 | 0.005 | (0.251,1.420) |
| **CONST** | – | -0.095 | 0.674 | (-0.541,0.350) | 0.325 | 0.112 | (-0.076,0.725) |

**Supplementary Table 4 Discrete Choice Experiment Education Subgroup Results Analysis.**

| **Characteristic factors** | **Level** | **Elementary School** | | | **Middle School and Above** | | |
| --- | --- | --- | --- | --- | --- | --- | --- |
|  |  | **β** | **P** | **95%CI** | **β** | **P** | **95%CI** |
| **Content of care** | Life care is the mainstay | – | – | – | – | – | – |
|  | Medical care | 0.394 | 0.053 | (-0.005,0.793) | 0.732 | 0.001 | (0.284,1.181) |
| **Average per capita cost** | – | -0.001 | 0.872 | (-0.003,0.003) | -0.007 | ＜0.001 | (-0.011,-0.004) |
| **Nursing staff** | Family members | – | – | – | – | – | – |
|  | Registered Nurse | -0.015 | 0.956 | (-0.556,0.525) | -1.042 | 0.001 | (-1.660,-0.424) |
|  | Care Worker | -0.379 | 0.164 | (-0.912,0.154) | -1.154 | ＜0.001 | (-1.775,-0.533) |
| **Frequency of care** | 4 hours*7 times/week | – | – | – | – | – | – |
|  | 1 hour * 7 times / week | -0.181 | 0.521 | (-0.734,0.372) | 0.352 | 0.252 | (-0.250,0.954) |
|  | 2 hours * 1 time / week | -0.308 | 0.276 | (-0.861,0.246) | 0.072 | 0.818 | (-0.543,0.688) |
| **Mode of compensation** | Mixed benefits | – | – | – | – | – | – |
|  | Proportional reimbursement | 0.380 | 0.175 | (-0.169,0.930) | -0.030 | 0.928 | (-0.669,0.609) |
|  | Cash subsidy | 0.644 | 0.023 | (0.088,1.200) | 0.610 | 0.051 | (-0.004,1.224) |
| **CONST** | – | 0.047 | 0.828 | (-0.376,0.469) | 0.192 | 0.371 | (-0.228,0.611) |

**Supplementary Table 5 Discrete Choice Experiment ADL Subgroup Results Analysis.**

| **Characteristic factors** | **Level** | **ADL Normal** | | | **ADL Mild/Moderate Disability** | | | **ADL Severe Disability** | | |
| --- | --- | --- | --- | --- | --- | --- | --- | --- | --- | --- |
|  |  | **β** | **P** | **95%CI** | **β** | **P** | **95%CI** | **β** | **P** | **95%CI** |
| **Content of care** | Life care is the mainstay | – | – | – | – | – | – | – | – | – |
|  | Medical care | 0.614 | 0.066 | (0.404,1.269) | 0.619 | 0.014 | (0.123,1.115) | 0.479 | 0.045 | (0.011,0.947) |
| **Average per capita cost** | – | -0.006 | 0.008 | (-0.011,-0.002) | -0.002 | 0.230 | (-0.006,0.001) | -0.003 | 0.072 | (-0.007,0.001) |
| **Nursing staff** | Family members | – | – | – | – | – | – | – | – | – |
|  | Registered Nurse | -0.896 | 0.059 | (-1.826,0.034) | -0.093 | 0.783 | (-0.753,0.567) | 0.557 | 0.091 | (1.201,0.088) |
|  | Care Worker | -0.962 | 0.044 | (-1.898,-0.262) | 0.302 | 0.357 | (-0.945,0.340) | -1.006 | 0.002 | (-1.647,-0.365) |
| **Frequency of care** | 4 hours*7 times/week | – | – | – | – | – | – | – | – | – |
|  | 1 hour * 7 times / week | 0.153 | 0.725 | (-0.703,1.010) | 0.166 | 0.632 | (-0.512,0.843) | -0.178 | 0.583 | (-0.812,0.457) |
|  | 2 hours * 1 time / week | 0.256 | 0.563 | (-0.611,1.124) | 0.201 | 0.569 | (-0.491,0.893) | -0.646 | 0.051 | (-1.295,0.004) |
| **Mode of compensation** | Mixed benefits | – | – | – | – | – | – | – | – | – |
|  | Proportional reimbursement | -0.648 | 0.134 | (-1.495,0.199) | 0.003 | 0.082 | (-0.079,1.325) | 0.455 | 0.199 | (-0.239,1.148) |
|  | Cash subsidy | 0.618 | 0.152 | (-0.228,1.464) | 0.586 | 0.096 | (-0.105,1.276) | 0.831 | 0.015 | (0.163,1.499) |
| **CONST** | – | 0.129 | 0.717 | (-0.568,0.826) | -0.300 | 0.371 | (-0.959,0.358) | 0.274 | 0.190 | (-0.135,0.684) |

**Supplementary Table 6 Discrete Choice Experiment Daily Caregiver Subgroup Results Analysis.**

| **Characteristic factors** | **Level** | **Nanny** | | | **Children or Spous** | | | **No Fixed or Others** | | | |
| --- | --- | --- | --- | --- | --- | --- | --- | --- | --- | --- | --- |
|  |  | **β** | **P** | **95%CI** | **β** | **P** | **95%CI** | **β** | **P** | | **95%CI** |
| **Content of care** | Life care is the mainstay | – | – | – | – | – | – | – | – | | – |
|  | Medical care | 0.434 | 0.139 | (-0.141,1.010) | 0.569 | 0.006 | (0.162,0.976) | 0.775 | 0.041 | (0.032,1.519) | |
| **Average per capita cost** | – | -0.005 | 0.026 | (-0.009,-0.001) | -0.004 | 0.018 | (-0.006,-0.006) | -0.002 | 0.443 | | (-0.008,0.003) |
| **Nursing staff** | Family members | – | – | – | – | – | – | – | – | | – |
|  | Registered Nurse | -0.429 | 0.301 | (-1.242,0.384) | -0.209 | 0.439 | (-0.736,0.319) | -1.782 | 0.002 | | (-2.901,-0.663) |
|  | Care Worker | -0.287 | 0.474 | (-1.075,-0.500) | 0.679 | 0.012 | (-1.211,-0.146) | -1.695 | 0.001 | | (-2.731,-0.658) |
| **Frequency of care** | 4 hours*7 times/week | – | – | – | – | – | – | – | – | | – |
|  | 1 hour * 7 times / week | 0.079 | 0.851 | (-0.741,0.899) | 0.037 | 0.893 | (-0.502,0.576) | 0.177 | 0.715 | | (-0.771,1.125) |
|  | 2 hours * 1 time / week | -0.274 | 0.509 | (-1.085,0.538) | 0.049 | 0.862 | (-0.506,0.604) | -0.593 | 0.257 | | (-1.618,0.433) |
| **Mode of compensation** | Mixed benefits | – | – | – | – | – | – | – | – | | – |
|  | Proportional reimbursement | -0.063 | 0.881 | (-0.897,0.769) | 0.176 | 0.539 | (-0.384,0.735) | 0.562 | 0.272 | | (-0.440,1.564) |
|  | Cash subsidy | 0.302 | 0.473 | (-0.524,1.128) | 0.591 | 0.033 | (0.048,1.134) | 1.202 | 0.023 | | (0.168,2.235) |
| **CONST** | – | 0.183 | 0.387 | (-0.231,0.596) | -0.098 | 0.704 | (-0.601,0.406) | 0.735 | 0.177 | | (-0.331,1.801) |
